# Discrepancies Between Patient‐Reported Discomfort and Clinical Documentation in Postoperative Care of Central Venous Catheters After Surgery: A Prospective Descriptive Study

**DOI:** 10.1002/hsr2.71632

**Published:** 2025-12-17

**Authors:** Maria Lithner, Adina Noghi, Thomas Kander

**Affiliations:** ^1^ Department of Health Sciences Lund University Lund Sweden; ^2^ Department of Surgery Skane University Hospital, Malmo, Sweden; ^3^ Department of Clinical Sciences Lund University Lund Sweden; ^4^ Department of Intensive and Perioperative care Sweden

**Keywords:** central venous catheter, CLABSI, device removal, documentation, infection control, postoperative care, vascular access device

## Abstract

**Background and Aim:**

Central venous catheters are vital in the peri‐ and postoperative period for patients undergoing abdominal surgery but are associated with risks such as catheter related infections. Signs of infection, like tenderness and induration at the insertion site, are important to recognize, as they may precede catheter‐related infections, but are often neglected. The aim was to compare surgical patients' experience of central venous catheter with the nursing documentation.

**Methods:**

Data was collected from short patient interviews and by reviewing electronic health record documentation.

**Results:**

In total, 106 patients were included, 103 of whom had a central venous catheter located in the internal jugular vein. The median (interquartile range) number of days with a catheter was 10 (8–16). There were no catheter related infections, but potential risks were identified. In 56% of the patients, central venous catheters were removed within the last 2 days before discharge. The most common reason for keeping the catheter was blood sampling. The results from the interviews indicate that 37 of the patients experienced considerable discomfort, such as persistent sensations of constriction, restricted mobility and pain, however, these symptoms were documented in the electronic health record for only three patients.

**Conclusion:**

The findings emphasize the necessity of systematically addressing patients' symptoms and individual needs in the postoperative care of the central venous catheter. Incorporating these aspects as a mandatory element in the standardized documentation template within the electronic health record will constitute an important first step toward enhancing patient‐centred care. There is a need for clearly defined guidelines outlining the indications for retention or removal of central venous catheters to minimize catheter‐days during postoperative care in surgical wards.

**Trial Registration:**

As this was a descriptive, qualitative observational study that did not involve testing a new intervention, compare interventions, or randomize participants, we did not find trial registration necessary under commonly accepted guidelines for clinical trial registration.

## Summary

- Central venous catheters are essential in surgical care but carry risks such as infections. Patient‐reported symptoms are often undocumented.

- Interviews revealed that patients reported considerably more catheter‐related symptoms and discomfort than were noted in the nursing documentation. Additionally, over half of the patients retained the catheter until one or 2 days prior to discharge.

- Including patient‐reported symptoms in the documentation template is an important first step toward patient‐centred care. Clear guidelines are also needed to determine when and why to retain or remove a central venous catheter.

**Abbreviations**
CRI: catheter related infections
CVC: central venous catheter
EHR: electronic health record
ICU: intensive care unit

## 1 Introduction

Central venous catheters (CVCs) play a crucial role in the perioperative care of surgical patients. CVCs are used for hemodynamic monitoring, the administration of vasopressors, intravenous infusions including parenteral nutrition and blood sampling [1]. However, the insertion and use of CVCs also carry certain risks. Short term complications can arise at the time of insertion, whereas catheter‐related infections (CRIs) are the most common and significant long‐term complications as they have been shown to increase mortality and health care costs [2, 3].

In previous studies on CRI, the main focus has been on critically ill patients, but the majority of CRIs have emerged in hospital wards outside the Intensive Care Unit (ICU) [4, 5]. Furthermore, no specialist training is required for registered nurses in general wards, and the patient‐per‐registered nurse ratio is multiplied several times compared with that in the ICU, making patient safety and patient‐centered care challenging [5, 6, 7].

Daily assessment of signs of infection, such as emergence of tenderness and induration at the insertion site, is important as these signs may precede the CRI [1, 8]. To identify early signs of infection, it is essential that nurses engage in active communication and attend to patients' self‐reported symptoms and early sensations of discomfort related to the CVC.

In summary, there is a paucity of knowledge on how well the documentation of discomfort from the CVC is performed by registered nurses in general wards. To further investigate this, we performed the current study with the aim to compare surgical patients' experience of CVC with the nursing documentation.

## 2 Methods

### 2.1 Design

This prospective clinical study had a descriptive design, including both qualitative data from patient interviews and quantitative data from the electronic health record (EHR).

### 2.2 Sampling and Recruitment

All patients with CVC at three general surgical wards at Skane University Hospital were consecutively asked for inclusion from March 2018 to January 2019. The exclusion criterion included the inability to read and sign the written study information in Swedish.

### 2.3 Data Sources

All CVC insertions were performed according to local guidelines, including a periprocedural checklist [3], and documented via an insertion template in the patients' EHRs at our department, as previously described [9]. The daily nursing care of patients with a CVC was documented in the same EHR system using a dedicated template that included standardized terminology related to CVC care ‐ such as daily inspection of the insertion site and dressings, documentation of any complications, and assessment on the ongoing need for the CVC or the reason for its removal. Swedish national guidelines recommend this information to be documented daily in the EHR. In addition, nurses were able to add free‐text notes to include information not covered by the standardized fields. The documentation template did not contain any section addressing patients' experiences with the CVC.

The nurses responsible for documentation in the EHRs in this study were registered nurses with a bachelor's degree in nursing.

Data on patients' perspectives of having a CVC were collected through brief, individual bedside interviews conducted in the patients' rooms on the surgical ward during the postoperative period. The interviews took place at a median of 3 days after surgery (range: 1–15 days). Three research nurses, not involved in direct patient care, conducted the interviews using two semi‐structured questions and recorded the patients' responses in real time through handwritten notes. The first question was open‐ended to capture both positive and negative patient experiences, while the second one was more probing to ensure that no adverse experiences were overlooked. *How do you experience having a catheter on the side of your neck?* and *Do you experience any discomfort from it?* As the interviews were conducted during the early postoperative period, patients were clearly affected by the recent surgery. The research nurses were mindful of this and approached the interviews with particular sensitivity. No field notes were used.

The first author (ML) was one of the study nurses, and also extracted all data from the EHR after the patients were discharged from the hospital. The responses from the individual bedside interviews were compared with data extracted from EHRs which had been documented by the anesthesiologists who inserted the CVCs and the nurses responsible for the care of patients with CVCs. No systematic assessment of data saturation was performed, as this descriptive study aimed to capture a broad range of perspectives and the inclusion of 106 patients was considered sufficient to achieve saturation.

### 2.4 Data Analysis

#### 2.4.1 Text Analysis

Content analysis was used to analyze the text with patient comments. Content analysis involves varying definitions and steps for interpreting the data. In the present study, the conventional approach, where the aim is to describe a phenomenon where the literature is limited, was used [10]. The advantage of this method is that information is collected directly from study participants without the use of preconceived categories and theories.

The answers from the patient interviews were read several times by all three authors (ML, AN and TK). Sentences or pieces of the text related to the aim of the study were grouped into six subcategories and two main categories by ML and AN. In the last step, all three authors reflected on the findings and agreed on the categories covering how patients perceived their experiences of having a CVC postoperatively. In the reporting of the results, a quantitative section was added to clarify how common this opinion was.

### 2.5 Sample Size and Statistics

The number of available patients during the study period was used to determine the sample size. No hypothesis tests were performed; only descriptive statistics were used. All continuous variables were presented as medians (ranges) and numbers were presented as percentages in parenthesis.

**Figure 1:**
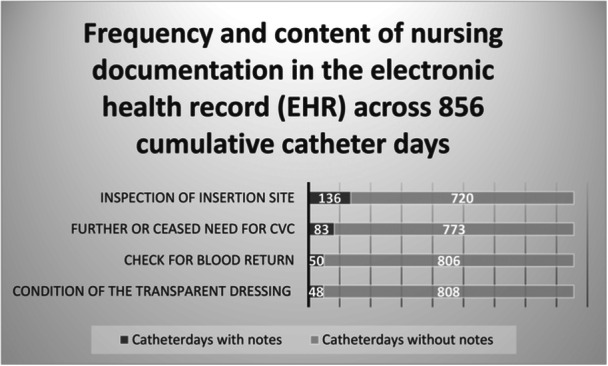
Frequency and content of nursing documentation in the electronic health record (EHR) across 856 cumulative catheter days.

**Figure 2:**
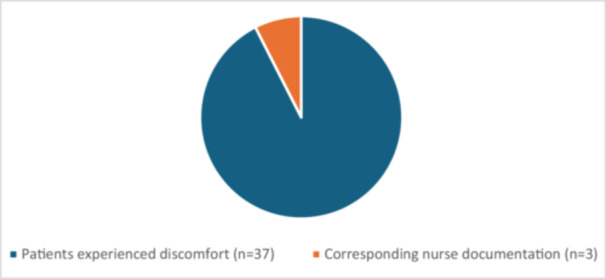
Discrepancy between patients' reported CVC discomfort and nursing documentation.

Microsoft Excel för Microsoft 365 MSO, version 2502 was used for compilation and processing of patient data in Table 1 and Figures 1 and 2.

**Table 1:** Patient characteristics.

Patients total, *n* (%)	106 (100)
Gender, female, *n* (%)	45 (42)
Age, years, median (interquartile range)	69 (63–75)
Main diagnosis, *n* (%)	
Malignancy	101 (95)
Other	5 (5)
Type of surgery	
Abdominal	105 (99)
Other	1 (1)
Length of hospital stay, days, median (interquartile range)	13 (10–19)
CVC insertion site, *n* (%)	
Right jugular vein	96 (91)
Left jugular vein	7 (7)
Right subclavian vein	1 (1)
Missing	2 (2)
Number of lumens, *n* (%)	
2	87 (82)
3	10 (9)
5	1 (1)
Missing	8 (7)
Catheter material, *n*	*n *= 97
Polyurethane	94
Silicone	3
Use of real time ultrasound guidance, *n* (%)	96 (91)
Number of CVC per patient, *n*	
1	101
2	4
3	1
Days with catheter, median (interquartile range), [sum]	10 (8–16) [856]
CVC removal: days before discharge, *n* (%)	
0	35 (33)
1	24 (23)
2	14 (13)
3	5 (5)
4	6 (6)
5‐28	7 (7)
Transferred to other hospital with CVC	10 (9)
CVC without removal date	5 (5)

### 2.6 Ethical Considerations

The study was approved by the Swedish Ethical Review Authority (2017/548), and all included patients provided written informed consent. The manuscript was written in accordance with the STROBE Statement for observational studies [11].

### 2.7 Participants

In all, 111 patients were invited to participate in this prospective study, and 106 were included in the final analysis (Table 1). The dropout in the study consisted of five patients; one patient who declined participation, one patient whose CVC was removed early, and three patients who were excluded because of administrative reasons.

## 3 Findings

In total 101 (95%) of the participants received a CVC in preparation for elective abdominal surgery related to malignancy in the esophagus, liver, pancreas, colon or rectum (Table 1). The CVCs were mostly placed in the jugular vein (*n* = 103) and 87 of the patients received two lumens. Five patients received more than one CVC due to postoperative complications and a prolonged stay in the intensive care unit. For two study patients, documentation of CVC‐insertion was lacking in the EHR. The total number of catheter days was 856 (Table 1).

The majority of the entries made by the nurses in the EHR were documented in the standardized CVC template (Figure 1). In addition, there were 10 patients with notes of free text in the EHR, describing abnormalities in the patients' CVC status. These 10 notes focused on redness at the insertion site and pain or discomfort indicated by the patients. In four of these entries, the nurse acted, such as changing the dressing for one patient and offering a sedative for anxiety and discomfort for another patient.

Interviews were conducted with 105 participants, one patient had the CVC removed prior to the interview. For details, please see Table 2. In summary, 57 patients appreciated the CVC and understood its benefits, such as avoiding peripheral venous catheters and blood sampling from peripheral veins. Some patients reported minor inconveniences, such as difficulties with clothing and feeling personal accountable for monitoring the CVC but generally accepted these as part of the treatment and recovery process. However, 37 patients articulated more pronounced discomfort, including persistent sensations of constriction, restricted mobility and pain from their CVC. Corresponding documentation of CVC‐related discomfort in the EHR was found for only three of these 37 patients (Figure 2).

**Table 2:** The postoperative patient experience of central venous catheter (*n* = 105).

Main categories	Subcategories	Patients (*n* = 105)	Quotation (Patient's study number)
Appreciating the benefits while accepting certain inconveniences	Demonstrating involvement and comprehension of the benefits	57	*No problem, nice to avoid blood samples from the arm. One needs to ensure that the infusion line goes with you (1)* *Only positive, is available for healthcare staff. Comfortable, considerate towards me as a person, makes it a lot easier (2)* *Compared to catheters everywhere, it is a relief. Do not have to think about how I move my hands. As good as it gets. Will demand one like this next time (3)*
	Managing activities of daily living	6	*A little more difficult to get dressed (4)* *Difficult to get on and off t‐shirts, avoids it and only wear cardigans (5) The bandage tightens and makes it difficult to move my head. Difficult to wear with the headscarf (6)*
	Feeling personally accountable for monitoring the CVC	5	*Many tubes to keep track of (7)* *Must keep an eye on the infusion lines (8)*
Expressing physical and emotional complaints	Persistent sensations of constriction, restricted mobility and pain	37	*Getting in the way, becoming clumsy. Needs to be better suspended and attached (9)* *Tightens across the neck and a bit difficult to breathe. Cannot sleep on that side as the infusion lines get in the way, it is truly distressing (6)* *Do not like it, hurts a lot. The infusion line pulls a lot (10)* *Had a lot of pain in the shoulders after surgery, worst on the right side where the infusion line was (11)* *The pump beeps depending on the position of the infusion line. The infusion line lay over my throat at night. The fixing tape comes off when you move (12)*
	Experiencing itching and cutaneous irritation beneath the dressing	5	*Got large rashes from the bandage that itched. Never reacted to bandages before (13)*
	Revealing distress and a diminished sense of attractiveness	5	*Experienced it as very unpleasant. Unpleasant thought of a needle in the throat. Asked for it to be removed, which was done (11)* *Tightens a bit, I feel ugly, unpleasant to look at (14)*

For 59 patients, the CVC was retained until the last day in the hospital or the day before discharge (Table 1). The nursing part of the EHR frequently mentioned delaying CVC removal, with entries such as “*Wait to remove the CVC. Blood samples are to be taken on Thursday”* or “*CVC can be removed tomorrow after blood samples are taken.”*

No confirmed CRIs were identified in this study. One patient developed sepsis 5 days after rectal surgery. Cultures from five blood samples, including the CVC, peripheral blood and one catheter tip, showed growth of the same bacteria, *Serratia marcescens*. However, the infection was assessed by the attending surgeon as being related to a surgical complication rather than CVC‐related complications. Notably, no nursing documentation was found regarding the condition of the CVC dressing or the insertion site for this patient, despite an initial suspicion of CRI.

## 4 Discussion

In this prospective observational study, patient‐reported experiences were compared with corresponding nursing documentation in the EHR regarding observations and interventions related to CVC care. The results are mainly based on CVCs in the internal jugular vein and demonstrate that most patients tolerate the CVC and understand and appreciate its benefits. However, a large group of patients experience considerably discomfort, such as persistent sensations of constriction, restricted mobility and even pain from the CVC. As patients experience multiple sources of postoperative discomfort and pain such as surgical incisions, drains and other catheters [12]—symptoms related to the CVC might be underrepresented in the nursing documentation within the EHR. The CVC were often kept for an extended period after surgery, and the majority of the patients still had the CVC the day before discharge from the hospital.

This study also gathered data from the EHRs provided by the anesthesiologists who inserted the catheter and the nurses from the surgical wards. The observed discrepancy between patient‐reported experiences and the corresponding documentation in the EHR, indicate that the documentation does not adequately reflect patients' perceived needs. However, it cannot be ruled out that a discrepancy existed between the needs identified by the nurse and what was documented in the EHR. The documentation template in the EHR used in this study did not capture patients own experiences, highlighting an opportunity to enhance patient involvement through the integration of such information into the template. This could potentially increase the inclusion of this type of documentation within the EHR.

Increasing the number of nursing interventions that need to be documented in the EHR may encourage nurses to perform the critical inspection of CVC during each shift, thereby improving patient safety. The EHR can also incorporate reminder functions to prompt nurses to document specific information every work shift [13]. However, studies indicate that a higher volume of electronic documentation may contribute to workflow fragmentation, reducing efficiency and potentially increasing the risk of burnout among healthcare professionals [14, 15]. To truly enhance patient safety while minimizing documentation burden, nurses need to perceive the documentation as valuable and the system as user‐friendly [14, 16]. This suggests that the systems are designed for ease of use and that healthcare organizations have prioritized providing nurses with training opportunities prior to implementation.

No confirmed CRIs were observed in this study, which may be attributed to the small sample size. However, potential risks of infection were identified. When the EHR of one patient with sepsis and surgical complications was studied, there was no nursing documentation concerning the state of the CVC insertion site. This could still mean that an inspection was carried out but was not documented. Overall, few notes in the nursing documentation described the condition of the insertion site or patients' experiences with the CVC. The limited documentation that exists tend to focus on the deviation from the norm, such as when patients actively report pain or when signs of redness at the insertion site are observed, potentially indicating a clinical risk. Inspection and palpation of the area closest to the CVC site is quick and easy to perform and can, for example, lead to earlier identification of an exit site infection [8]. It is therefore both warranted and important to incorporate this alongside patient‐reported symptoms in routine preventive CVC care, rather than considering it solely in the context of identifying a primary infection focus during manifest sepsis.

A considerable number of patients in the study reported discomfort associated with their CVC, Table 2. Some participants characterized the sensation as:

Tightens across the neck and a bit difficult to breathe. Cannot sleep on that side as the infusion lines get in the way, it is truly distressing.

(Patient nr 6)

Do not like it, hurts a lot. The infusion line pulls a lot.

(Patient nr 10)

The exact source of this discomfort, whether related to catheter positioning or the applied dressing, remains uncertain. Ensuring an effective dressing is essential not only for maintaining catheter integrity but also for optimizing patient comfort, particularly when the patient is in an upright position. Failure to maintain an intact dressing is one of the most frequently reported breaches in CVC care in both ICU and surgical wards [17], and with an ambulatory patient, it becomes more difficult. The discomfort and pain from the pulling of the catheter can be reduced to some extent by carefully fixing the tubes from the CVC or making a slight change in the exterior position of the CVC while changing the dressings. The addition of tissue adhesive can improve the durability of the dressing but also increases the risk of skin irritation [18]. Patients who have sensitive skin and experience discomfort from the dressing can benefit from skin protection under a semipermeable dressing and the altering of dressing material.

To minimize patient discomfort from a CVC, it is challenging for the anesthesiologists to find both the safest and the most comfortable position for the CVC. Previous studies have shown that CVC‐insertion and postprocedural comfort are better with a subclavian approach than with an internal jugular approach, while also reducing the risk for CRI [19, 20, 21]. However, it also slightly increases the risk for pneumothorax, making the risk‐benefit assessment challenging [21]. According to some authors, the anticipated need for a CVC for more than 7 days justifies the subclavian route over the internal jugular route [22]. For a vast majority of patients in the current study the total number of days with CVC was more than 7 days, indicating a change towards subclavian CVCs in this cohort.

More than half of the patients in this study maintained their CVC until the last 2 days in the hospital. The daily need assessment of the CVC was part of the documentation template in this study but was rarely used. Preventing CRIs is a complex and long‐lasting undertaking that includes several different components of various importance. Focusing on the maintenance phase following insertion, early removal of the CVC clearly reduces the risk of catheter related infection [4, 23] and thus constitutes a key target for continued quality improvement.

Nursing documentation suggests that considerations such as avoiding unnecessary or challenging peripheral venous access may influence the decision to retain the CVC. Even though a reduction in the number of CVC days also reduces the frequency of CRIs, it still needs to be balanced with the risk associated with the insertion of other intravascular catheters such as peripheral venous catheters or midline catheters, to replace the CVC [23, 24]. To reduce the number of CVC‐days, there is a clear need for guidelines with explicit indications to guide the decision to retain or remove the postoperative CVC. An expert panel using the Delphi technique presented a foundation for decision making concerning CVCs [25]. For example, such guidelines need to include the number of CVC‐days, what the CVC is currently being used for, the quality of the patient's peripheral veins, the type of medications and infusions administered and the site condition, including any patient‐reported symptoms. This information is critical to support daily clinical decision‐making regarding CVC management during interprofessional team rounds in surgical wards.

### 4.1 Strength and Limitations of the Study

A key strength of this study lies in the triangulation of perspectives through data collection from patients, anesthesiologists and nurses in the care unit, as well as the inclusion of three separate surgical wards.

There are some limitations to this study that should be considered. First, only EHR data were used to describe the care provided by nurses and anesthesiologists, which could have been complemented by direct observations for a more comprehensive understanding. Second, due to the patients' postoperative condition, the interviews were kept short and focused rather than long and in‐dept, which may have limited the exploration of all aspects of discomfort related to the CVC. Patients may have provided overly positive responses to the questions in order to appear satisfied and appreciative toward the interviewers. As a result, it cannot be guaranteed that all relevant dimensions of discomfort related to CVC were fully addressed during the interviews. Third, no formal method was used to assess data saturation in relation to the interview material. However, as 105 patients were interviewed similar responses recurred several times and thereby reinforcing the findings. Forth, as all patients were able to communicate with the interviewer, the transferability is limited to communicative surgical patients. Fith, given the single‐center design, the generalizability of the findings to other healthcare settings may be limited and should be interpreted with this context in mind.

## 5 Conclusion

In conclusion, the findings highlight the value of integrating patient‐reported symptoms and perceived discomfort associated with CVC into postoperative care practices in surgical wards.

## Author Contributions

**Maria Lithner:** conceptualization, data curation, formal analysis, funding acquisition, investigation, methodology, project administration, supervision, validation, writing – original draft, writing – review and editing. **Adina Noghi:** formal analysis, methodology, validation, writing – review and editing. **Thomas Kander:** formal analysis, funding acquisition, methodology, supervision, validation, writing – review and editing.

## Disclosure

The lead author Maria Lithner affirms that this manuscript is an honest, accurate, and transparent account of the study being reported; that no important aspects of the study have been omitted; and that any discrepancies from the study as planned (and, if relevant, registered) have been explained.

## Consent

Informed consent to publish analyses of patient data was obtained from participants at the time of inclusion. Data supporting the findings of this study are available upon reasonable request from the corresponding author. The study was approved by the Swedish Ethical Review Authority (2017/548), and all included patients provided written informed consent.

## Conflicts of Interest

The authors declare no conflicts of interest.

## Recommendation for Future Research

Future research should focus on establishing evidence‐based guidelines to clarify the clinical indications for retaining or removing CVC following surgery.

## Data Availability

Data available on request from the authors.
